# Whey Protein Isolate-Xylose Maillard-Based Conjugates with Tailored Microencapsulation Capacity of Flavonoids from Yellow Onions Skins

**DOI:** 10.3390/antiox10111708

**Published:** 2021-10-27

**Authors:** Ștefania Adelina Milea, Iuliana Aprodu, Elena Enachi, Vasilica Barbu, Gabriela Râpeanu, Gabriela Elena Bahrim, Nicoleta Stănciuc

**Affiliations:** Faculty of Food Science and Engineering, Dunarea de Jos University of Galati, 111 Domnească Street, 800201 Galați, Romania; adelina.milea@ugal.ro (Ș.A.M.); iuliana.aprodu@ugal.ro (I.A.); elena.ionita@ugal.ro (E.E.); vasilica.barbu@ugal.ro (V.B.); gabriela.rapeanu@ugal.ro (G.R.); Gabriela.Bahrim@ugal.ro (G.E.B.)

**Keywords:** glycation, flavonoids, microencapsulation, onion skins, antioxidant activities

## Abstract

The objective of this study is to encapsulate flavonoids from yellow onion skins in whey protein isolates (WPI) and xylose (X), by Maillard-based conjugates, as an approach to improve the ability to entrap flavonoids and to develop powders with enhanced antioxidant activity. WPI (0.6%, *w*/*v*) was conjugated to X (0.3%, *w/v*) through the Maillard reaction at 90 °C for 120 min, in the presence of a flavonoid-enriched extract. Two variants of powders were obtained by freeze-drying. The glycation of WPI allowed a better encapsulation efficiency, up to 90.53 ± 0.29%, corresponding to a grafting degree of 30.38 ± 1.55%. The molecular modelling approach was used to assess the impact of X interactions with α-lactalbumin and β-lactoglobulin on the ability of these proteins to bind the main flavonoids from the yellow onion skins. The results showed that X might compete with quercetin glucosides to bind with α-lactalbumin. No interference was found in the case of β-lactoglobulin. The microstructural appearance of the powders revealed finer spherosomes in powder with WPI–X conjugates via the Maillard reaction. The powders were added to nachos, followed by a phytochemical characterization, in order to test their potential added value. An increase in antioxidant activity was observed, with no significant changes during storage.

## 1. Introduction

The recent trends in food intakes have experienced a transition to a more healthy oriented nutrition, which shifted eating habits to natural foods, paving the way for the extraction and identification of new biologically active compounds, with beneficial or even therapeutic functions, with a considerable emphasis put on well-being and the prevention of disease. Therefore, obtaining and incorporating bioactive-enriched plant extracts in food may significantly contribute to lowering the risk of specific illnesses [1]. Certain advantages may result from the use of bioactive-enriched plant extracts when compared with individual or synthetic compounds, particularly in terms of the synergistic actions of different molecules [2].

Onion (*Allium cepa* L.) is cultivated around the world, being the second most grown horticultural crop after tomatoes. It has been estimated that more than 550,000 tonnes of onion skin bio-waste was generated by the use of the 89 million tonnes onion harvest [3]. The onion waste represents an environmental problem since it is not suitable for animal feeding and so is usually sent to landfill. Onion skins and the outer layers contain significant quantities of fiber and phenolic compounds, such as flavonoids, glucosides, phenolic acids, and organosulfur compounds [3]. In particular, the onion solid waste is rich in quercetin, quercetin glucosides, quercetin polymers, ferulic acid, gallic acid, and kaempferol, with significant beneficial effects [4] associated with biological activities such as: antidiabetic, antioxidant, anti-inflammatory, anticancer, antimicrobial, and enzyme inhibitory effects [5]. Thus, it can be appreciated that onion has nutritional complexity and holds suitable potential for functional food development, as a source of antioxidant, antimicrobial, anticancer, and antibrowning compounds [6].

Nowadays, the food industry is focusing on implementing methods for the valorization of onion solid waste, as a natural resource with a high amount of value-added ingredients, into eco-friendly functional foods [7]. However, adding polyphenols in a free form in foods may lead to chemical instability due to the unsaturated bonds contained in their molecular structures. The stability is affected by the presence of oxidants, heat, light, and enzymes during storage [8]. Suitable techniques to protect phenolic compounds from chemical damage before their industrial application carry out microencapsulation using different methods, such as freeze-drying [9], which may overcome the drawbacks of their instability, improve their bioavailability as well as shelf life [10] and widen the industrial applications in the food, pharmaceutical and cosmetics industries [11]. In our recent study, different delivery systems were developed for extracts enriched in onion skin flavonoids using a unique combination of whey protein isolates, whey proteins hydrolysates, pectin, and maltodextrin as coating materials [12]. The coating materials should have thermal or mechanical stability to protect the core materials from external factors. Since proteins have amphiphilic properties, they can correlate with the interaction of various chemical groups. However, when using proteins as coatings, some limitations should be considered, given by several external factors, including pH variation, ionic strength and in vitro proteolysis by pepsin, which lead to the degradation of protective walls, causing the release and degradation of bioactives during digestion [13]. These authors tested various structural designs, such as Maillard-based conjugation, to modify the structure and properties of whey proteins and to produce more stable delivery systems with excellent properties. The functional and physico-chemical properties gained with glycation reaction refer to significantly improved emulsifying properties, thermal stability, antioxidant properties, antibacterial activity, and water solubility [14], simultaneously with enhancing the thermal stability of proteins over a wide range of pH and thermal aggregation values [13]. Numerous studies are focused on whey protein as an encapsulating material, but, to the best of our knowledge, there are no studies using whey protein isolates (WPI) in conjugate form with xylose (X) as an encapsulation material for flavonoids. Therefore, the aim of this study was to test the possibility of using WPI–X Maillard-based conjugates as coating materials for flavonoids extracted from yellow onions skins. Flavonoids were isolated by means of solid–liquid extraction in combination with ultrasound-assisted extraction using ethanol as solvent. The WPI–X conjugates were generated via heating in an alkali environment, whereas the flavonoid microcapsules were generated using freeze-drying. Two powders were obtained, using WPI-X conjugates with and without heating, and the resulting freeze-dried powders were characterized in terms of their encapsulation efficiency, phytochemical content and antioxidant activity. Structural and morphological particularities of the samples were analyzed using confocal laser electron microscopy. In order to test the added value, the powders were added to the recipe of a food product (nachos), followed by phytochemical characterization. The results obtained in this study could bring certain benefits in terms of exploiting the bioactive potential of phytochemicals and glycation reaction for developing formulas with improved functional properties.

## 2. Materials and Methods

### 2.1. Chemicals

Whey protein isolate (WPI) (protein content of 95%) was purchased from Fonterra (New Zealand). Xylose (X) (about 99% purity) and the reagents used to determine the total phenolic compounds, total flavonoid content, 2,2-diphenyl-1-picrylhydrazyl (DPPH), and 6-hydroxy-2,5,7,8-tetramethylchroman 2-carboxylic acid (Trolox) were purchased from Sigma-Aldrich Corp. (St. Louis, MO, USA). All other reagents were of analytical grade.

### 2.2. Ethanolic Ultrasound-Assisted Extraction of Flavonoids from Onion Skins

Yellow onions were purchased from a local market (Galati, Romania) in June 2020. The outer layers of onions were collected, cleaned with distilled water and dried. Before extraction, in order to obtain a homogeneous batch of particle size, the onion skins were ground to sizes smaller than 0.5 mm × 0.5 mm and used for further extraction. The flavonoidic extract was obtained by mixing 50 g of the onion skins with 450 mL of 70% ethanol solution and glacial acetic acid (ratio 9:1, *v*/*v*). The extraction was performed using a sonication bath at 40 °C for 30 min. In order to obtain flavonoids-enriched extracts, the extraction was repeated three times, while the supernatants were centrifuged at 5000× *g* for 10 min at 4 °C, collected and concentrated under reduced pressure at 40 °C. The obtained extract was characterized in terms of selected phytochemicals and used for microencapsulation experiments.

### 2.3. Preparation of WPI–X–Flavonoid Conjugates

WPI–X conjugates were prepared according to the method described by Jia et al. [13] with minor modifications. An amount of 60 mg/mL of WPI was first dissolved in 100 mL ultrapure water under gentle stirring, followed by the addition of 30 mg/mL of X (mass ratio of 2:1). The mixture was allowed to hydrate for 14 h at room temperature (25 °C). After complete hydration, about 750 mg of concentrated flavonoid-enriched extract was added and the mixture was allowed to dissolve by ultrasonication for 1 h at 35 °C. The resulting solution was divided into two: variant 1 (coded V1) and variant 2 (coded V2). The pH of V2 solution was adjusted at 9.0 and placed in a sealed screw-top glass tube. In order to promote the Maillard conjugation, Variant 2 was heated to 90 °C in water bath for 3 h. After heating, the temperature of Variant 2 was lowered to 25 °C in an ice bath. Both variants were freeze-dried (CHRIST Alpha 1–4 LD plus, Osterode am Harz Germany) at −42 °C under a pressure of 10 Pa for 48 h and stored at −4 °C.

### 2.4. Characterization of the Extract and of Microencapsulated Powders

Both the extract and the powders were characterized for flavonoids, total phenolic compound contents and antioxidant activity using the aluminum chloride method, Folin–Ciocâlteu and DPPH method, respectively, as described by Milea et al. [12]. The total flavonoid contents are expressed in mg quercetin equivalents (QE)/g dry weight (DW), whereas the total polyphenol contents are expressed in mg gallic acid equivalents (GAE)/g DW. In each case, the concentrations of bioactives and antioxidant activity were expressed through selected standard calibration curves.

The encapsulation efficiency of the powders was calculated as described by Saénz et al. [15]. In brief, the microencapsulation efficiency was determined by assessing the surface flavonoid contents (SFC) and total flavonoid contents (TFC) of the powders, expressed as mg QE/g DW. In order to quantify the SFC, 50 mg of powders was mixed with 5 mL of ethanol and methanol (ratio 1:1, *v*/*v*). These dispersions were stirred at room temperature for 1 min and then centrifuged at 4000× *g* and 4 °C for 10 min. For TFC, 50 mg of powder was accurately weighed and dispersed in 5 mL of a mixture of ethanol, acetic acid, and water (50:8:42, *v*/*v*/*v*). The resulting dispersion was vortexed (1 min), followed by ultrasonication for 30 min at 40 ± 1.0 °C, to break the microcapsules. The supernatant was centrifuged at 14,000× *g* for 10 min and then filtered. The content of flavonoids in the resulting supernatants was measured by the aluminum chloride method, as explained by Milea et al. [12]. The microencapsulation efficiency (ME, %) was calculated using Equation (1):

(1) $$\mathrm{ME}\;\left(\%\right)=\frac{\mathrm{TFC}-\mathrm{SFC}}{\mathrm{TFC}}\times 100$$

The antioxidant activity was assessed using the DPPH method. Briefly, 0.1 mL of supernatant resulted from TFC determination was added to 3.9 mL of DPPH stock solution. The DPPH stock solution was prepared mixing 3 g of DPPH with 100 mL of methanol. Simultaneously, a control sample was prepared by adding 0.1 mL methanol to 3.9 mL DPPH. The absorbance for both samples was read at 515 nm after 1.5 h. The results were calculated using a calibration curve and are expressed in mMol Trolox/g DW.

### 2.5. Browning Index and Grafting Degree Measurement of the Powders

The browning intensity was measured using a spectrophotometric method with minor modifications [16]. A volume of 5 mL of a mixture consisting of equal volume of acetic acid (2% *v*/*v*) and formaldehyde (1% *v*/*v*) was added to 0.1 mL of 1 mg/mL of samples and centrifuged for 10 min at 4500× *g*. The resulting supernatant (5 mL) was mixed with 5 mL of ethanol and the mixture was centrifuged again. Absorbances of the supernatant at 420 and 600 nm were measured. The difference between the two absorbance values was used to evaluate the browning index.

The grafting degree was determined using the o-phthalaldehyde (OPA) method, according to Jia et al. [13]. A volume of 4 mL of OPA was added to 0.2 mL of the diluted samples (0.1 mg/mL) in test tubes. Upon homogenization, all tubes were placed in a water bath at 35 °C for 1 min. The absorbance was measured at 340 nm. A blank sample was also made using the same volume of ultrapure water. Grafting degree was determined using Equation (2):

(2) $$\mathrm{Grafting}\;\mathrm{Degree}\;\left(\%\right)=100\times \frac{A_{0}-A_{s}}{A_{0}}$$

where A_0_ and A_s_ are the absorbance of blank sample and absorbance of tested samples, respectively.

### 2.6. In Silico Investigations

In agreement with the experimental approach, the molecular modeling tools were used to simulate X binding by the two major whey proteins. The three-dimensional molecular models of the α-lactalbumin (α-LA, PDB ID: 1F6S) [17] and β-lactoglobulin monomers (β-LG, PDB ID: 4DQ3) [18] from the RCSB Protein Data Bank were optimized and relaxed at 25 °C using GROMACS 4.6 software [19], in agreement with the protocol previously described by Aprodu et al. [20]. The equilibrated protein models were used as receptors for the X binding. The PatchDock algorithm [21], which is very efficient for performing protein–small ligand docking, was used to identify the most probable binding site of X molecules to the WP. Matching the receptor and ligand molecules was carried out through rigid body docking, which is based on the shape complementarity principles. The algorithm employed involves the following major stages: the surface of the receptor was first segmented to identify the so-called hot spot residues on the concave, convex or flat geometric patches, which were selected for a further surface patch matching step with the ligand. The resulting complexes were filtered to disqualify the solutions, involving steric clashes, and finally ranked based on the geometric shape complementarity score. The best three WPI–X fits were selected based on the binding energy values among the potential docking models generated by the PatchDock algorithm [21]. An in-depth analysis of the binding pockets was carried out using the PDBePISA [22] tools and DoGSiteScorer web server [23] to identify the extent to which protein glycation affects flavonoids binding.

### 2.7. Confocal Laser Microscope Spectroscopy

A confocal laser scanning microscopy analysis of the samples was employed to assess the structural appearance of the microencapsulated powders. The CLSM images were captured with a Zeiss confocal laser scanning system (LSM710) equipped with several types of lasers such as a diode laser (405 nm), Ar laser (458, 488, 514 nm), DPSS laser (diode pumped solid state—561 nm) and HeNe laser (633 nm). The powders were observed with a 20× apochromatic objective, at zoom values of 1 and 0.6, respectively. The obtained 3D images were rendered and processed by ZEN 2012 SP1 software (Black Edition).

### 2.8. Formulation of a Value-Added Food Product

To support the multifunctional properties, the powders were added as an ingredient in a nacho recipe at a ratio of 3%. The recipe involved mixing of corn (250 g) and wheat flour (100 g) in a ratio of 2.5:1, onion (100 mg), pepper (10 mg), oil (20 mL), salt (10 mg), powders (3%) and water (150 mL). The corresponding samples were coded as N1 and N2. The control sample was nachos without the addition of microencapsulated powder (C). Samples were homogenized and allowed to stand for 1 h at room temperature to equilibrate. After homogenization, the nachos were formed and cooked for 6 min in an oven (3 min on each side) at 200 °C. The storage stability of bioactives was tested at 0 days and after 28 days at 25 °C.

### 2.9. Statistical Analyses

All analyses were performed in triplicate and data are reported as mean ± standard deviation (SD). After running the normality and homoscedasticity tests, experimental data were subjected to one-way analysis of variance (ANOVA) in order to identify significant differences. The Tukey method with a 95% confidence interval was employed for post hoc analysis; *p* < 0.05 was considered to be statistically significant. The statistical analysis was carried out using Minitab 18 software.

## 3. Results

### 3.1. Phytochemical Characterization of the Yellow Onion Skin Extract

The solid–liquid ethanolic ultrasound-assisted method applied in this study allowed us to obtain a bioactive-enriched extract, containing flavonoids of 228.7 ± 3.0 mg QE/g DW, with a total polyphenolic content of 96.1 ± 2.7 mg GAE/g DW, and yielding an antioxidant activity of 495.9 ± 2.4 mM TEAC/g DW. In our previous study, different extraction techniques were tested in order to select the most suitable method to obtain flavonoid-enriched extracts from yellow onion skins [24]. The results showed a satisfactory content in phytochemicals, when comparing the ultrasound-assisted technique versus the conventional solid–liquid extraction. However, the selection of the ultrasound-assisted extraction in this study was based on the reduction time and protection of thermolabile compounds. Therefore, Constantin et al. [24] reported similar values for flavonoid contents in ultrasound-assisted extracts of 230.6 ± 8.4 mg QE/g DW. Additionally, Milea et al. [12] extracted the biologically active compounds from yellow onion skins using a similar method and reported flavonoids of 97.3 ± 3.0 mg QE/g DW, polyphenols of 55.3 ± 2.5 mg GAE/g DW and an antioxidant activity of 345.0 ± 2.7 mM TE/g DW. Singh et al. [25] used an ultrasound-assisted method to extract the bioactive compounds from onions. The extraction with 70% ethanol showed similar values for flavonoid extraction of 212.3 ± 14.6 mg QE/g and a higher amount of phenolic compounds (418.0 ± 34.4 mg GAE/g). On the other hand, Pobłocka-Olech et al. [26] extracted flavonoids from different varieties of yellow onion skins using only methanol and obtained a lower level compared with the current results, between 2.4 and 12.2 mg QE/g. Benito-Román et al. [27] performed a comparative study of polyphenols from onion wastes between conventional and ultrasound-assisted extraction. They reported smaller values for flavonoid contents, ranging from 7.7 ± 0.1 to 23.8 mg QE/g dry onion skins (DOS). The different values could be explained by the distinct selected parameters, different origin of raw materials or by the method of expressing final results (DW/DOS). Likewise, the experimental conditions allowed us to extract a significant amount of polyphenols (73.3 ± 1.8 mg GAE/g DOS), which were further increased to 102.1 ± 5.1 mg GAE/g DOS.

The difference between results is due to the extraction method. As is known, ultrasound-assisted method simplifies and accelerates the extraction because the high-intensity ultrasounds increase pressure and temperature, causing a disruption of the cell wall of the matrix, with the subsequent release of polyphenols. Moreover, this technique offers the advantages of lower extraction times and temperatures compared to conventional extraction techniques [28].

### 3.2. Correlation between Browning Intensity, Grafting Degree and Microencapsulation Efficiency

An effective method to improve the functional properties of proteins, including the ability to include and protect low molecular bioactives, is based on the interaction with polysaccharides and smaller carbohydrates, via Maillard conjugation [29]. The Maillard reaction is a complex reaction occurring between amines and carbonyls [30], which involves, first, to consumption of the free amino group by the carbonylation reaction, mainly coming from the free amino group on the side chain such as lysine and arginine, or the free amino group on the N-terminus of the peptide chain of the protein molecule. Therefore, the carbonyl condensation between the reducing sugar and nucleophilic amino group could be analyzed by the loss of amino acids in the reaction [31]. Ideally, as reported by Jiménez-Castanõ et al. [32], to produce a glycol conjugate destined for incorporation into food, to avoid the formation of the highly colored, insoluble, nitrogen-containing polymeric compounds, referred to as melanoidins, the Maillard reaction needs to be performed under carefully controlled conditions to prevent the later stage changes. As reported by Jia et al. [13], protein glycation by the Maillard reaction might be favored in alkali conditions, while glycation is inhibited by the partial denaturation of the protein in acid conditions. These authors suggested a higher grafting degree and lower browning intensity at pH 9.0 after heating for 3 h. Therefore, these parameters were selected in our study to promote Maillard-based conjugates between WPI and X.

The brown-colored pigment formation in foods is caused by the Maillard reactions or caramelization [16]; therefore, the browning index is generally accepted as an indicator of the Maillard reaction. The brown pigment formation in the microencapsulated powders was evaluated by absorbance measurements at 420 nm and 600 nm, respectively. As expected, the browning intensity was higher (0.12 ± 0.01) for the heat-treated variant (Variant 2) than for the untreated variant (0.09 ± 0.01) (Variant 1). A proportional increase was observed between the browning intensity and antioxidant activity of the powders, indicating the strong antioxidant potency of the glycated variant due to the heating process. The powders showed significant differences in antioxidant activity (*p* < 0.05), with values of 179.7 ± 4.5 mMol TE/g DW for V1 and 184.4 ± 0.7 mMol TE/g DW for V2. Suminar et al. [33] explained that this was probably caused by reducing sugar reacting more easily with amino acids in heating conditions and producing antioxidant activity. The formation of the conjugate was also confirmed by grafting degree, which is able to reflect the level of glycation [13]. In the present study, a correlation between the grafting degree and encapsulation efficiency can be observed in both variants. Thereby, a grafting degree of 22.6 ± 2.5% and an encapsulation efficiency of 86.7 ± 1.4% were observed for V1. A significantly higher (*p* < 0.05) values were estimated for Variant 2, with a grafting degree of 30.4 ± 1.6% and an encapsulation efficiency of 90.5 ± 0.3%. Therefore, it can be appreciated that the Maillard-based conjugates showed a higher ability to entrap the flavonoids from yellow onion skin extract. These results indicate that the glycated form of the powder has a positive effect on the encapsulation efficiency.

In the conditions applied in our study, the Maillard-based conjugates between WPI and X caused structural changes in proteins that improved the ability to entrap flavonoids, in good agreement with reports of Liu et al. [34], Xu et al. [35] and Liu et al. [36]. The glycation degree can be correlated with the decrease in available -NH_2_ groups. For example, Shang et al. [37] suggested a dramatic loss in Lys and Arg, whereas a significant decrease in Tyr and Cys was also found, due to the formation of the dehydroalpropyl side chain. These authors also reported a transition toward a higher molecular weight distribution of WPI heated in the presence of X, at 90 °C and 95 °C and pH 9.0, whereas the contents of protein polymers larger than 40 kDa increased with the reaction time, thus indicating a protein crosslinking phenomenon. The heat-induced glycation reaction between WPI and X molecules might induce the formation of hydrogen bonds, thus weakening the interaction between molecules, and result in a reduction in the β-sheet and β-turns but an increase in the random coil [37].

In another study, lycopene was encapsulated in whey protein isolate and xylo-oligosaccharides conjugates and presented values ranging from 10.0 ± 0.4% to 27.0 ± 0.5%, depending on the other parameters of the reaction (temperature, time, pH). Muhoza et al. [38] evaluated the possibility of glycating the casein by the Maillard reaction with dextran for delivering coenzyme Q_10_. These authors reported that when the reaction time was less than 8 h, the grafting degree of the mixture about 20%. Ghatak and Iyyaswami [39] encapsulated quercetin from dry onion peels and obtained an encapsulation yield between 40.4% and 96.4%. They found that the highest encapsulation yield was achieved under the following process conditions: casein concentration of pH 7.09 for the crude extract containing the quercetin concentration of 16.27 M. Akdeniz et al. [40] reported the encapsulation efficiency values of phenolic from onion skins as being between 55.6 and 89.2% for different coating material combinations, with a maximum value found for maltodextrin:casein in a ratio of 6:4.

### 3.3. Molecular Modeling

**Figure 1:**
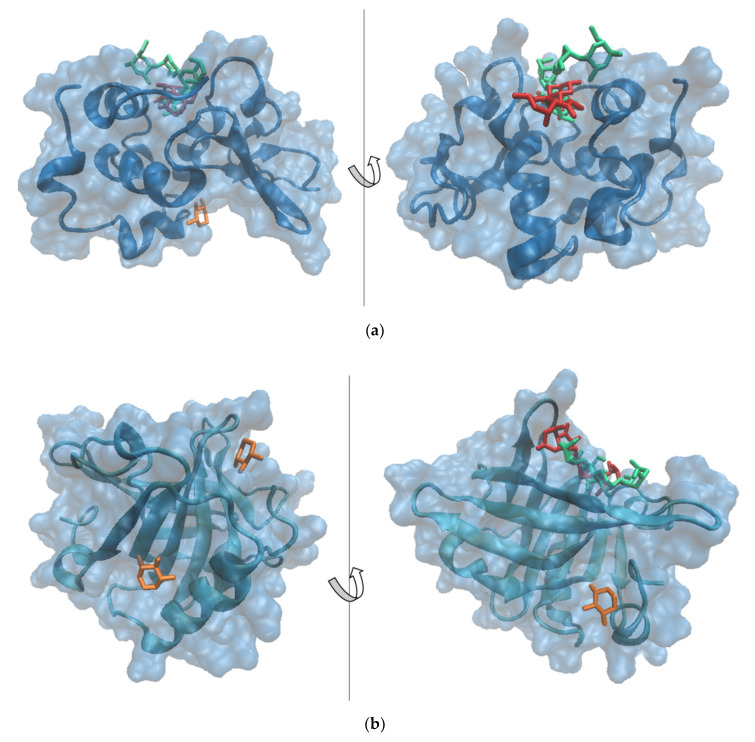
Superposition of the models showing the most probable complexes formed between (**a**) α-lactalbumin and (**b**) β-lactoglobulin (represented in blue in surf style) and xylose (represented in orange in licorice style—models 1 and 2), quercetin-4′-*O*-monoglucoside (represented in red in licorice style) and quercetin-3,4′-*O*-diglucoside (represented in green in licorice style). Images were prepared using VMD software [42].

Details on how protein glycation with X impacts further flavonoids binding were collected by means of an in silico approach. The potential binding sites for X were predicted for both α-LA and β-LG molecules by performing molecular docking simulations. The best three fits involving α-LA and β-LG as receptors, decided based on the binding energy values and the interface area, were further analyzed in detail (Table 1). In the case of α-LA, mainly two binding sites located on the protein surface appeared to accommodate the ligands used in the study. The highest affinity of the receptor for its ligand was estimated based on the lowest binding energy. Thus, α-LA exhibited the highest affinity toward X molecules when bound to the cavity involving residues of the α-LA core (Phe^53^, Gln^54^, Tyr^103^, Trp^104^) and the amino-terminal section of the Leu^105^-Leu^110^ helix. In addition to the hydrophobic contacts, three hydrogen bonds of 3.68 Å, 2.37 Å, and 2.83 Å involving Thr^33^, Leu^105^ and Ala^106^, respectively, contributed to the attraction between α-LA and the X molecule hosted within this cavity. Two different relative binding positions of the X molecules with respect to the α-LA receptor, sharing common amino acid residues in contact with the ligand, were predicted with high scores. None of the two X binding modes appeared to affect the attachment of the main flavonoids prevailing in the onion skin extract, namely quercetin-4′-*O*-monoglucoside (QMG) and quercetin-3,4′-*O*-diglucoside (QDG) [41], to the α-LA. In agreement with Horincar et al. [41], the α-LA molecule accommodates, with high specificity, the same binding site of both QDG and QMG ligands (Figure 1). The amino acids establishing direct contacts with QMG are Leu^3^, Glu^11^, Leu^12^, Lys^13^, Asp^14^, Thr^38^, Leu^52^, Leu^85^, Thr^86^, Asp^88^, Ile^89^, Met^90^ and Lys^93^, whereas the residues responsible for the QDG binding are Glu^1^, Leu^3^, Arg^10^, Glu^11^, Leu^12^, Lys^13^, Thr^38^, Leu^52^, Asp^83^, Leu^85^, Thr^86^, Asp^88^ and Ile^89^ [41].

In addition, this wide pocket with a volume of 435.8 Å^3^ appears to be able to accommodate an X molecule, which overlaps the QMG without interfering with QDG binding. It should be noted that α-LA shows a better affinity towards QMG and QDG (binding energy of −24.21 kcal/mol and −32.01 kcal/mol, respectively) with respect to X (binding energy of −7.48 kcal/mol).

On the other hand, the β-LG molecule is able to accommodate the X molecule in three different pockets with volumes ranging from 137.86 to 217.15 Å (Table 1), without interfering with QMG or QDG binding (Figure 1).

β-LG binds the X molecules more tightly compared to α-LA; the X binding energy by the β-LG monomer varies between −16.41 and −13.00 kcal/mol. In addition, the binding of two X molecules to the β-LG pockets involves hydrogen bonds established with Glu^158^ in the case of complex 1 and Met^24^, Asp^137^ and Leu^149^ in the case of complex 2, as presented in Table 1. The in silico results successfully complement the experimental findings, adding valuable details on how whey protein glycation with X further impacts flavonoid binding. These atomic level observations indicate that, upon glycation, the β-LG molecule might play a major role in flavonoid biding.

### 3.4. Phytochemical Profile of the Powders

The two powders showed a total flavonoid content of 97.7 ± 3.7 mg QE/g DW in V1 and 120.0 ± 1.6 mg QE/g DW in V2. Total polyphenolic content showed no significant (*p* < 0.05) differences of 45.1 ± 0.6 mg GAE/g DW and 46.9 ± 1.5 mg GAE/g DW in V1 and V2, yielding a corresponding antioxidant activity of 179.7 ± 4.5 mMol TE/g DW and 184.4 ± 0.7 mMol TE/g DW, respectively. Therefore, the glycation of WPI allowed a better encapsulation of flavonoids, yielding a powder with a higher antioxidant activity. To the best of our knowledge, no other studies are available that exploit the potential of WPI conjugates as biopolymeric wall materials used in the microencapsulation of flavonoids.

In a previous study, Milea et al. [43] encapsulated flavonoids from yellow onion skins using maltodextrin, pectin and whey protein hydrolysates as coating materials in different ratios. The concentration of flavonoids, polyphenols and the antioxidant activity of the freeze-dried variants showed comparable levels, as flavonoids varied from 98.1 ± 0.5 to 103.7 ± 0.6 mg QE/g DW, whereas significant higher polyphenol contents (varying from 53.5 ± 1.7 to 69.3 ± 1.0 mg GAE/g DW) and antioxidant activities (varying from 280.6 ± 3.1 to 337.6 ± 0.9 mM TE/g DW) were reported for different variants. Horincar et al. [41] used different combinations of biopolymeric coatings based on whey protein isolate and chitosan, maltodextrin and pectin as adjuvants for encapsulation. These authors obtained two variants of freeze-dried powder with different profiles. Therefore, lower values for total flavonoid content of 5.8 ± 0.2 mg QE/g DW and antioxidant activity of 175.9 ± 1.5 mM TE/g DW were suggested in coatings with WPI-chitosan. When using a more complex biopolymeric wall material, including WPI-maltodextrin-pectin, these authors obtained a powder with significant higher flavonoid content and antioxidant activity of 104.9 ± 5.0 mg QE/g DW and 269.2 ± 3.6 mM TE/g DW, respectively.

### 3.5. Structure and Morphology of the Powder

The confocal laser scanning microscopy technique allows the simultaneous identification of several compounds, at the surface of the particles using specific samples that usually discharge light at different wavelengths. This type of analysis also permits the visualization of the internal morphology of any type of particle by different fluorophores labels, hence displaying the compositional evolution of the targeted molecules, which represents the main aim of any study that regards the protection of valuable molecules such as antioxidant compounds, mainly polyphenols, through an encapsulation process. Therefore, it can be applied as a nondestructive visualization technique for microparticles.

**Figure 2:**
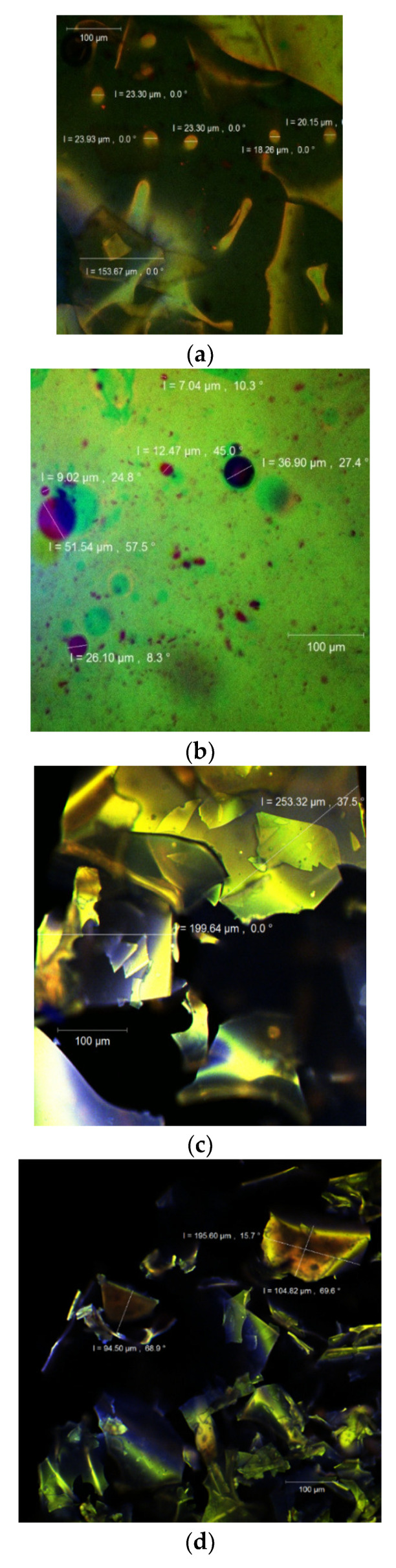
Confocal laser scanning microscopy images of the V1 sample ((**a**)—native state and (**b**)—stained with fluorophores) and V2 sample ((**c**)—native state and (**d**)—stained with fluorophores).

By using a confocal laser scanning Carl Zeiss 710 microscope with the ZEN 2012 SP1 software (Black Edition), the images of V1 and V2 powders (Figure 2) were captured, both in the native state without any other additional dye added (Figure 2a,c) and stained with Congo Red (Figure 2b,d, respectively). In the native state, the powders showed an autofluorescence (in the range of 520–580 nm) due to the rich content of polyphenols among which quercetin predominates [39]. The biologically active compounds were captured in the WPI–X matrix (with a displayed autofluorescence showed in blue). Several irregular scaly formations could be observed with larger dimensions for the V2 (199.6–253.3 µm), compared to V1 (94.5–104.8 µm), probably due to the WPI–X conjugates. The microstructure was rather similar to that reported by Horincar et al. [41], who used different polymers as the encapsulating matrices such as chitosan, maltodextrin and pectin.

Through the fluorescent staining with Congo Red, a fluorophore usually used to highlight the fluorescence of proteins, several spherosomes were revealed, highlighting the encapsulated flavonoids in the WPI–X matrix. Nonetheless, the displayed formations were larger (up to 51.5 µm) and less numerous in the V1 sample where a significant amount of nonencapsulated flavonoids was visualized (Figure 2b).

The interaction of the WPI with the small carbohydrates via the Maillard reaction favored a better incorporation of the biologically active compounds from the onion extract into finer spherosomes (approximately 20 μm) or in the form of scales with digitiform extensions. The fluorophore bound to the conjugated proteins and generated a fine, orange wall with a fluorescent emission between 600 and 620 nm around the bioactives.

### 3.6. Characterization of New Formulated Food Product

To test selected functionality, the powders were added to a recipe of nachos in a ratio of 3%. Therefore, three variants of nachos were obtained according to the two variants, coded as N1 (3% addition of variant V1) and N2 (3% addition of variant V2) and a blank without powder (C). The obtained food products were analyzed in terms of bioactive stability for 28 days over storage at 25 °C. As expected, the differences in bioactives and antioxidant activity between samples correlated with the added quantity of powder (Table 2).

During the storage test, no significant decrease (*p* > 0.05) was found in the flavonoid contents of N1, in contrast with N2, where a significant decrease of 9% was observed (*p* < 0.05). From Table 2, a significant increase in antioxidant activity values for both variants, during storage, can be observed, probably due to the release of some other compounds, apart from flavonoids, such as phenolics from microcapsules. Therefore, an increase in antioxidant activity was found in both variants, at approximatively 26%. Milea et al. [12] reported a decrease of 43% for flavonoids, 35% for polyphenols and 8% for antioxidant activity, in the case of a new formulated soft cheese with the addition of 1% microencapsulated powder, and 47% for flavonoids and 31% and 9% for polyphenols and antioxidant activity in the case of a soft cheese with 2% microencapsulated powder.

## 4. Conclusions

In this study, flavonoid-loaded microcapsules using whey protein isolates glycated with xylose via the Maillard reaction were successfully obtained. The liquid–solid, ultrasound-assisted extraction method was applied to obtain a flavonoid-enriched extract. The extract showed a significant content of flavonoids and a satisfactory antioxidant activity. Whey protein isolates and xylose were used, in nonglycated and glycated forms, as possible candidates for the microencapsulation of flavonoid-enriched onion skin extracts by freeze-drying. Both powders were characterized, showing significant amounts of polyphenols, flavonoids and a remarkable antioxidant capacity. A positive correlation was found between the browning index and antioxidant activity, and consecutively between the grafting degree and microencapsulation efficiency. The confocal laser scanning microscopy confirmed the higher ability of the whey protein isolate–xylose conjugates to entrap flavonoids. The molecular docking studies allowed the identification of the potential zones from α-lactalbumin and β-lactoglobulin surfaces involved in the interaction with xylose molecules. Xylose appeared to attach with high affinity to the α-lactalbumin protein pocket involved in flavonoid binding. In the case of β-lactoglobulin, the tested ligands docked to different sites; smaller cavities located on the protein surface are preferred by xylose. The powder was added to nachos, and a slightly decrease in phytochemicals was found during storage. However, the antioxidant activity of the added-value products increased, probably due to the release of some other bioactives from microcapsules. Based on the reported results, the protein–monosaccharide Maillard-type conjugates are a good alternative for food ingredient carriers and promising attractive methods of delivery.

## Figures and Tables

**Table 1:** Molecular details on the interactions between the main whey proteins (α-lactalbumin (α-LA) and β-lactoglobulin monomer (β-LG)) equilibrated at 25 °C, xylose (X) and the major flavonoids from onion skins (quercetin-4′-*O*-monoglucoside (QMG) and quercetin-3,4′-*O*-diglucoside (QDG) [41].

	α-LA—X			α-LA—QMG	α-LA—QDG
	Complex 1	Complex 2	Complex 3	Horincar et al. [41]	
***Interaction descriptors***					
Amino acids interacting with ligands	Thr^33^, Glu^49^, Phe^53^, Gln^54^, Tyr^103^, Trp^104^, Leu^105^, Ala^106^	Thr^33^, Val^42^, Asn^44^, Glu^49^, Phe^53^, Gln^54^, Tyr^103^, Trp^104^, Leu^105^, Ala^106^	Glu^11^, Leu^12^, Asp^14^, Leu^15^, Thr^38^, Leu^85^, Thr^86^, Ile^89^, Met^90^, Lys^93^	Leu^3^, Glu^11^, Leu^12^, Lys^13^, Asp^14^, Thr^38^, Leu^52^, Leu^85^, Thr^86^, Asp^88^, Ile^89^, Met^90^, Lys^93^	Glu^1^, Leu^3^, Arg^10^, Glu^11^, Leu^12^, Lys^13^, Thr^38^, Leu^52^, Asp^83^, Leu^85^, Thr^86^, Asp^88^, Ile^89^
Binding energy, kcal/mol	−13.00	−10.52	−7.48	−24.41	−32.01
Interface area, Å^2^	153.5	147.5	159.8	625.20	541.20
***Pocket descriptors***					
Volume, Å^3^	339.58	339.58	381.50	435.84	
Depth, Å	15.72	15.72	13.18	13.94	
Enclosure	0.16	0.16	0.20	0.30	
	β-LG—X			β-LG—QMG	β-LG—QDG
	Complex 1	Complex 2	Complex 3	Horincar et al. [41]	
***Interaction descriptors***					
Amino acids interacting with ligands	Tyr^20^, Tyr^42^, Val^43^, Glu^44^, Gln^59^, Cys^66^, Pro^126^, Leu^156^, Glu^157^, Glu^158^, Gln^159^, Cys^160^, His^161^	Ala^23^, Met^24^, Ala^25^, Leu^133^, Phe^136^, Asp^137^, Leu^140^, Arg^148^, Leu^149^, Ser^150^	Ile^2^, Leu^93^, Glu^108^, Ala^111^, Gln^115^, Leu^117^	Ala^37^, Pro^38^, Leu^39^, Val^41^, Leu^58^, Lys^60^, Ile^71^, Ile^84^, Asp^85^, Ala^86^, Leu^87^, Asn^88^, Asn^90^, Met^107^, Glu^108^, Asn^109^	Pro^38^, Leu^39^, Val^41^, Leu^58^, Lys^60^, Glu^62^, Ala^67^, Lys^69^, Ile^71^, Asn^90^, Met^107^, Glu^108^, Asn^109^
Binding energy, kcal/mol	−13.37	−16.41	−13.00	−35.05	−34.37
Interface area, Å^2^	169.1	152.8	157.6	502.10	585.90
***Pocket descriptors***					
Volume, Å^3^	217.15	141.31	137.86	229.70	
Depth, Å	9.42	7.68	10.16	13.08	
Enclosure	0.12	0.19	0.19	0.33	

**Table 2:** Phytochemical profile of added-value nachos and stability during 28 days of storage.

Selected Phytochemicals	Control		Samples with 3% Addition of Variant 1		Samples with 3% Addition of Variant 2	
	Time 0	After 28 Days	Time 0	After 28 Days	Time 0	After 28 Days
Total flavonoids, mg QE/g DW	0.66 ± 0.02 ^a^	0.63 ± 0.02 ^a^	1.04 ± 0.05 ^a^	0.99 ± 0.01 ^a^	1.08 ± 0.05 ^a^	0.98 ± 0.04 ^b^
Total polyphenols, mg GAE/g DW	0.73 ± 0.04 ^a^	0.75 ± 0.02^2^	1.24 ± 0.03 ^a^	1.22 ± 0.05 ^a^	1.37 ± 0.19 ^a^	1.08 ± 0.019 ^a^
Antioxidant activity, mMol TE/g DW	156.07 ± 2.57 ^b^	197.97 ± 1.74 ^a^	157.89 ± 1.41 ^b^	199.49 ± 0.81 ^a^	158.19 ± 0.48 ^b^	198.57 ± 0.35 ^a^

Means on the same row that do not share letter (a, b) are significantly different, based on Tukey method and 95% confidence.

## Data Availability

Data are contained within the article.
